# Case Report: Molecular Diagnosis Revealing an Intestinal Infection of a Hybridized Tapeworm (*Taenia saginata* and *Taenia asiatica*) to Human in Yunnan, China

**DOI:** 10.3389/fgstr.2022.845850

**Published:** 2022-02-21

**Authors:** Kan-Kan Chu, Ye Qiu, Ce-Heng Liao, Zhi You, Zuo-Shun He, Wen Fang, Hong-Ying Li, Peter Daszak, Jun-Jie Hu, Yun-Zhi Zhang, Xing-Yi Ge

**Affiliations:** ^1^ Institute of Preventive Medicine, School of Public Health, Dali University, Dali, China; ^2^ Hunan Provincial Key Laboratory of Medical Virology, College of Biology, Hunan University, Changsha, China; ^3^ Dali Prefecture Institute of Schistosomiasis Control and Prevention, Dali, China; ^4^ EcoHealth Alliance, New York, NY, United States; ^5^ School of Biological Sciences, Yunnan University, Kunming, China

**Keywords:** human taeniasis_1_, hybridized tapeworm_2_, *Taenia asiatica*_3_, *Taenia saginata*_4_, molecular diagnosis_5_.

## Abstract

Human taeniasis caused by tapeworms is an intestinal disease prevalent in many countries around the world. *Taenia asiatica*, *Taenia saginata*, and *Taenia solium* are the most common pathogens causing human taeniasis. Among the three species of tapeworms, *T. saginata* and *T. asiatica* share high similarity in their genomes and have been reported to be capable of hybridization with each other. Here, we reported a case of an 18-year-old male patient hospitalized in Yunnan Province, China, in 2019. Due to long-lasting abdominal distension and white tapeworm segments in the feces, the patient was diagnosed with taeniasis. He was treated with traditional Chinese medicine, and a tapeworm approximately 2.7 m long was expelled. The morphology of the eggs and gravid proglottids of the tapeworm was observed. Interestingly, the tapeworm was identified as a hybrid between *T. saginata* and *T. asiatica* according to molecular and phylogenetic analyses. This case is the first documented case of human taeniasis caused by a *T. saginata* and *T. asiatica* hybrid in Yunnan Province. Molecular evidence suggests that the hybrid of *T. saginata* and *T. asiatica* may have caused widespread infection in rural areas of Western China, and further investigation and research on these parasites in Western China are needed. The method described in this case may be helpful for future research.

## Introduction

Human taeniasis is usually caused by three species of tapeworms in the genus *Taenia*, including *Taenia asiatica*, *Taenia saginata*, and *Taenia solium*. In the larval stage (cysticercus), *T. solium* and *T. asiatica* are mainly parasitic in the liver and muscle of pigs and wild boars. The cysticercus of *T. saginata* mainly lives in the striated muscle of cattle (1). These tapeworms have been reported to infect humans *via* ingestion of raw or undercooked contaminated pork or beef (2). *T. asiatica* is mainly confined to Asian countries including Korea, China, Thailand, Philippines, Indonesia, Vietnam, Japan, Lao PDR, Nepal, and India, while *T. saginata* and *T. solium* are distributed worldwide (3–9). Studies have shown that *T. saginata* and *T. asiatica* are very close in genetic relationship and do not form complete reproductive isolation (10, 11). Here, we report a rare case caused by a *T. saginata*–*T. asiatica* hybrid in Yunnan Province, China, 2019.

## Case Description

On February 3, 2019, an 18-year-old male was hospitalized in E’Shan, Yunnan, China, due to persistent abdominal distension and white tapeworm segments in the feces for 1 week. According to the survey, the patient frequently ingested barbecue beef and pork as his daily diet. A complete blood count and biochemical analyses were performed, and the results showed a high eosinophil count (5.3 × 10^8^/L). On the same day, traditional Chinese medicine was prescribed for treatment, with the initial oral administration of pumpkin (*Cucurbita moschata*) seed powder, followed by areca nut (*Areca catechu*) extract 1 h later, further followed by a 30% hydrated magnesium sulfate (MgSO_4_) solution half an hour later. A cestode approximately 2.7 m long was expelled 1.5 h after the medicine was administered. The patient has a good prognosis and was given medical advice to avoid raw and undercooked meat.

**Figure 1:**
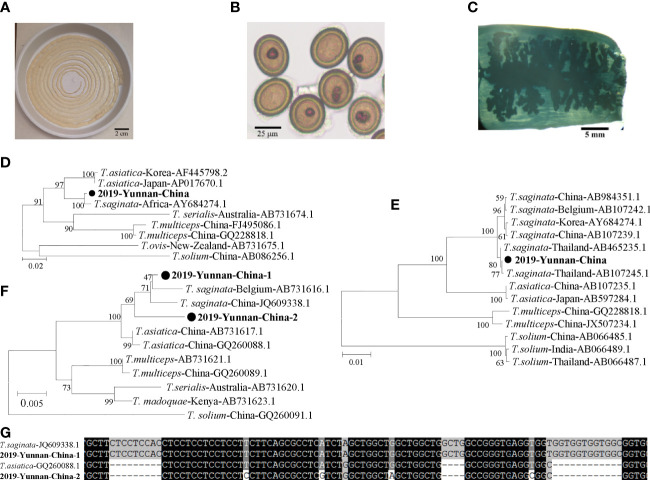
Human taeniasis caused by a *T. asiatica* and *T. saginata* hybrid in an 18-year-old boy in Yunnan, China, 2019. **(A)** The tapeworm recovered from the patient (approximately 2.7 m in length). **(B)** Eggs and proglottids of the tapeworm derived from feces. **(C)** A gravid proglottid showing main lateral branches. **(D)** Phylogenetic relationships between the nadh1 nucleotide sequences of the case and those of *T. asiatica*, *T. saginata*, *T. solium*, and *T. multiceps* from various countries. **(E)** Phylogenetic analysis of the *cox1* gene. **(F)** Phylogenetic analysis of the 2 different nucleotide sequences of the 18S rRNA gene obtained from the case. **(G)** Alignment of the short region in the 18S rRNA gene containing 2 deletions.

The tapeworm specimen was collected and preserved in a dish with phosphate buffer solution (**Figure 1A**). Eggs and gravid proglottids from the tapeworm were extracted from the fecal sample (**Figure 1B**). Tapeworm segments were stained, and >13 uterine segments were observed (**Figure 1C**). Proglottids of the tapeworm were used to extract genomic DNA using a TIANamp Genomic DNA kit (Tiangen, Beijing, China). The full-length mitochondrial cytochrome c oxidase 1 (cox1) gene, NADH dehydrogenase 1 (nadh1) gene, and nuclear 18S ribosomal RNA (18S rRNA) gene were amplified. The primers were designed as follows: Cox1F (5′-TTA GAG GAA ATT GTG AAG TTA CTG CT-3′) and Cox1R (5′-TTA TAA GAA TCC ACC AAG CAT GAT GC-3′) for cox1; Nadh1F (5′-CTC AGG AGA ACT CTT TAT GTG GAG C-3′) and Nadh1R (5′-CAC ACG ACT ATA ATG GTA CCT AAC-3′) for nadh1; and 18SF (5′-CTT CAC AGC CAC TGC TGC TAA CAC-3′) and 18SR (5′-TCC TGC CAG TAG TCA TAT GCT TGT CT-3′) for the 18S rRNA gene. All the replicons were ligated into T-vectors, and the full gene was sequenced.

The complete 912-bp *nadh1* gene sequences showed 99.2% nucleotide (nt) identity to *T. saginata*, 95.7%–95.9% identity to *T. asiatica*, and 87.1%–87.4% identity to *T. solium* (**Figure 1D**). The complete 1,620-bp *cox1* gene sequences showed 99.1%–99.9% nt identity to that of *T. saginata*, 95.7%–96.1% identity to that of *T. asiatica*, and 88.8%–89.1% identity to that of *T. solium* (**Figure 1E**). However, 2 different sequences of the 18S rRNA gene were identified, indicating two heterozygous alleles in the worm genome. One allele (2019-Yunnan-China-1) was 2,604 bp in length which was clustered into the same terminal branch with that of *T. asiatica*, while the other allele (2019-Yunnan-China-2) was 2,579 bp forming a separate branch parallel to those of *T. asiatica* and *T. saginata* (**Figure 1F**). As for the nt sequences, 2019-Yunnan-China-1 showed 99.4% identity to that of *T. saginata* and 97.8% identity to that of *T. asiatica*, while 2019-Yunnan-China-2 showed 98.3% identity to that of *T. asiatica* and 97.5% identity to that of *T. saginata*. The major different part among these sequences was amplified by PCR for further alignment which revealed that 2019-Yunnan-China-1 was the same as that of *T. saginata* except for only one nucleotide while 2019-Yunnan-China-2 was more similar to *T. saginata* (**Figure 1G**). This alignment suggested that the two alleles might originate from *T. saginata* and *T. asiatica*, respectively. In summary, *cox1* and *nadh1* indicated that this specimen was closest to *T. saginata* reported from Thailand, but the 18S rRNA gene indicated this tapeworm was a hybrid of *T. saginata* and *T. asiatica*.

## Discussion

Yunnan Province is located in the southwest region of China. In history, due to humid climate, home animal husbandry and some eating habits, taeniasis is common in Yunnan (12). With the improvement of sanitary conditions and the strengthening of health education, the traditional custom of eating raw pork has been gradually banned. However, taeniasis is still prevalent in some rural areas in Yunnan. A current investigation in 4 townships in Yunnan reported a 16.71% infection rate in the total population, and all the cases were diagnosed as *T. asiatica* infection (6). In this case, the possible source of this patient’s infection was ingestion of undercooked pork and beef, because the survey showed that this patient liked barbecue food which could be hardly cooked thoroughly, and their family has no habit of eating raw pork. Furthermore, we tested the patient’s parents for taenia infection, and the results were negative. Therefore, other than raw meat, undercooked meat should also be avoided to prevent taenia infection.

Recent studies reported several cases of hybridization between *T. saginata* and *T. asiatica* in Asia (13–16). In 2010, a research team identified hybridized tapeworms in taeniasis patients in Yajiang County of Ganzi Prefecture in Sichuan Province, by using a mitochondrial *cox1* gene and two nuclear genes (*ef1* and *elp*). In that case, the worm from one adult had the *cox1* gene homologous to *T. saginata* but both nuclear genes were heterozygous with *T. saginata* and *T. asiatica* alleles, while the worm from the other adult had *cox1* of *T. asiatica*, *elp* of *T. saginata*, and heterozygous e*f1* alleles of *T. saginata* and *T. asiatica* (16). Later, more hybrids of *T. asiatica* and *T. saginata* were identified by amplifying mitochondrial DNA (mtDNA) and nuclear DNA in Muli County, Sichuan Province (10). Thus, hybrid cases of *T. saginata* and *T. asiatica* might be widely distributed in rural areas of western China, although all reported cases were contained in Sichuan previously. In this study, we reported the first case of tapeworm hybrid infection in Yunnan, another province located in western China. Home animal husbandry keeping different animals together is common, but parasite control is usually omitted in rural areas in Yunnan, leading to high incidence of human taeniasis. The infection of hybrids of porcine and bovine taenia indicated potential interspecies transmission of taenia among the domestic animals near human communities, which might contribute to the prevalence of taeniasis. In order to effectively prevent and control parasitic infections, the publicity and popularization of public health knowledge is required. Meanwhile, a variety of measurements to improve animal husbandry, such as animal quarantine, laughter management, parasite monitoring, food quality supervision, and the related legislation, are suggested to control taeniasis. Nevertheless, further investigation on the pathogens of human taeniases in Yunnan is needed for more specific control and prevention advice.

The morphological characteristics of adult worms, larvae, and ova are used for cestode identification. However, phenotypic methods are time-consuming and require special expertise. PCR amplification sequencing and real-time PCR have been employed to determine cestode species (17). The targets are conserved regions in mitochondrial genes (*nadh1* and *cox1*) and ribosomal RNA genes (18S and 28S rRNA). Previous studies generally sequenced *nadh1* and *cox1*. However, our case indicated that sequencing the mitochondrial genes only may not be enough to identify hybridization between closely related species, such as *T. asiatica* and *T. saginata*, due to the matrilineal inheritance of mitochondrial genes (15). Thus, combining the genotypes of mitochondrial and rRNA genes together will enhance the reliability of species determination.

In all, this case study provides a pioneering view of pathogenic taenia in Yunnan which may promote the understanding about the pathogenesis of human taeniasis in western China and help the disease control. However, the only case can hardly reveal the whole picture of the diversity of pathogenic taenia in Yunnan for which further investigation is required.

## Data Availability Statement

The raw data supporting the conclusions of this article will be made available by the authors, without undue reservation. All the sequences were deposited in GenBank under accession numbers MN452861-MN452864.

## Ethics Statement

This research was approved by the Medical Ethics Committee of Dali University under number DLDXLL2018008 and was obtained with the informed consent of all participants. The patients/participants provided their written informed consent to participate in this study. Written informed consent was obtained from the individual(s) for the publication of any potentially identifiable images or data included in this article.

## Author Contributions

KKC and YZZ treated the patient and collected the sample. CHL, ZY, and YQ performed the gene amplification, cloning, sequencing, and analysis. ZSH, WF, and JJH performed the morphological investigation. XYG coordinated and designed the experiment. KKC, YQ, XYG, HYL, PD, and YZZ wrote the manuscript. All authors contributed to the article and approved the submitted version.

## Funding

This work was jointly funded by the National Natural Science Foundation of China (U2002218 and 81874274 ), and the Yunnan Health Training Project of High-Level Talent (L-2017027).

## Conflict of Interest

The authors declare that the research was conducted in the absence of any commercial or financial relationships that could be construed as a potential conflict of interest.

## Publisher’s Note

All claims expressed in this article are solely those of the authors and do not necessarily represent those of their affiliated organizations, or those of the publisher, the editors and the reviewers. Any product that may be evaluated in this article, or claim that may be made by its manufacturer, is not guaranteed or endorsed by the publisher.
